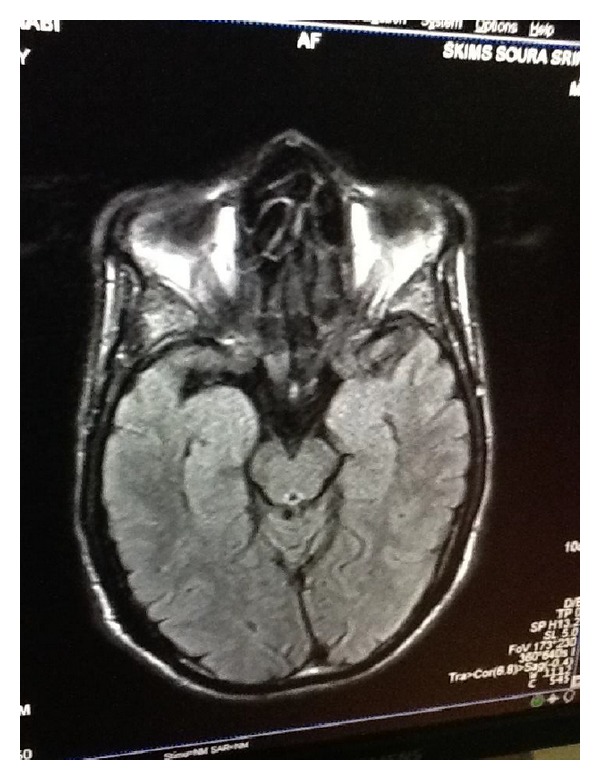# Unusual Presentation of Uncommon Disease: Anorexia Nervosa Presenting as Wernicke-Korsakoff Syndrome—A Case Report from Southeast Asia

**DOI:** 10.1155/2014/482136

**Published:** 2014-05-22

**Authors:** Raheel Mushtaq, Sheikh Shoib, Tabindah Shah, Mudasir Bhat, Randhir Singh, Sahil Mushtaq

**Affiliations:** ^1^Department of Psychiatry, Government Medical College, Srinagar, Kashmir, India; ^2^M.B.B.S, GMC Srinagar, Kashmir, India; ^3^Department of Radiology, Government Medical College, Srinagar, Kashmir, India; ^4^Department of Prosthodontics, GDC Srinagar, Kashmir, India; ^5^M.B.B.S, ASCOMS, Jammu, India

## Abstract

Anorexia nervosa presenting as Wernicke-Korsakoff syndrome is rare. The causes of Wernicke-Korsakoff syndrome are multiple like alcohol abuse, thyrotoxicosis, haemodialysis, severe malnutrition because of gastric carcinoma and pyloric obstruction, hyperemesis gravidarum, and prolonged parenteral feeding. We report a case of anorexia nervosa, who presented with Wernicke's encephalopathy and progressed to Korsakoff's syndrome. Knowledge, awareness, and early intervention of anorexia nervosa by mental health professionals can prevent development of Wernicke-Korsakoff syndrome.

## 1. Introduction

Carl Wernicke (1881) was the first to describe Wernicke's encephalopathy (WE) [1]. WE consists of a classical triad of symptoms that include ataxia, global confusion, and ophthalmoplegia [2]. The prevalence of Wernicke's encephalopathy ranges from 0.5 to 2.8 percent [1]. However, only about one-third of the patients report the classical triad of symptoms in Wernicke's encephalopathy [2]. Korsakoff's syndrome (KS) on the other hand is a memory disorder that presents with amnesia, confabulations, attention deficits, and disorientations [3]. The key features in Korsakoff's syndrome include the inability to learn new information or form new memories and the inability to retrieve old memories. Without thiamine supplementation, its deficiency can lead to WE and eventually can lead to permanent condition of Korsakoff's syndrome [3]. The various causes of Wernicke-Korsakoff syndrome include alcohol misuse, thyrotoxicosis, haemodialysis, severe malnutrition because of gastric carcinoma and pyloric obstruction, hyperemesis gravidarum, prolonged parenteral feeding, and hunger strike [4, 5]. Wernicke's encephalopathy is a neurological disorder due to deficiency of thiamine [1]. Thiamine is an essential vitamin necessary for various biochemical pathways in the brain. Thiamine depletion that lasts for 2-3 weeks can lead to varied brain lesions. The brain lesions occurs in vulnerable areas like diencephalon and brainstem [5]. There are just 3-4 cases of Wernicke's encephalopathy (WE) in patients with anorexia nervosa [6–8]. To the best of the investigator's knowledge, there are just few case reports, in which WE progressed to KS [5, 8]. This is the first case report from this part of the world (India) regarding anorexia nervosa, presenting as WE and progressing to KS subsequently.

## 2. Case Report

A 39-year-old unmarried Indian female smoker was brought to psychiatric consultation with complaint of inability to walk and confusion. She was running her family business at present and was a model when she was a teenager. She had abnormal behaviour in form of eating selective foods and history of recurrent thoughts about being fat, leading to severe restriction in her diet. On detailed history from the attendants, it was revealed that she had been dieting since she was 16 years old and her symptoms waxed and waned during this time period. The patient's aim was to weigh 30 kilograms (kg). As a result she would vomit major portion of the food items that she ate. At the age of 23, her food intake reduced drastically to pieces of cucumber, selective fruits, and a cup of yoghurt. She became more obsessed with her body shape and regularly measured her waist circumference and checked her body weight as well. At the age of 36, she developed amenorrhea and had lost 10 kilograms (kg) of weight over the last few years in order to reach her ideal weight of 30 kgs. However the patient refused to seek any psychiatric consultation in the past. Based on the detailed history, she was subsequently diagnosed as anorexia nervosa as per ICD-10 [9]. But she was not taking any treatment for this disorder. There was no significant medical history in the past. Physical examination revealed asthenic female, with a body temperature of 98.6°F. Neurological examination revealed nystagmus, ptosis, pupils unreactive to light, upward gaze palsy of the left eye, and gait ataxia. During intraoral examination, there were generalised smooth glossy erosions on the palatal surfaces of maxillary teeth and soft palate was found to be erythematous. During our evaluation, the patient denied mood disturbance and auditory/visual hallucinations or delusions; however, she did confabulate many stories. She was not oriented to time, person, and place. Short-term memory was not intact, as she was unable to remember three items after few minutes, even with cue recognition. Her insight of her illness was poor and she scored 16/30 on minimental state examination (MMSE). Complete blood count, serum glucose, blood urea, and serum creatinine and electrocardiogram (ECG) were within normal limits. Electrolytes were normal except potassium (2.2 meq/L), and magnesium levels were decreased (0.43 mg/dL). Folic acid was 2.1 ng/mL (NR = 3.2–17.0 ng/dL) and B12 was 250 pg/mL (NR = 202–900 mg/dL). Urinary analysis, chest X-ray, and liver function test were also found to be normal.

**Figure 1:**
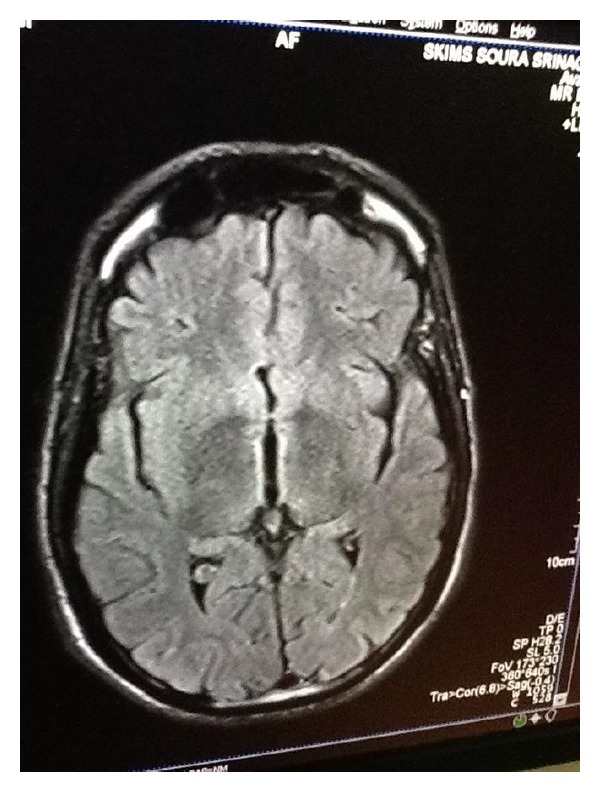


The important radiological findings on MRI brain in patients include lesions in mammillary bodies, thalamus, and tectal plate and hyperintensities in the periaqueductal area. These radiological findings represent typical lesions characteristic of WE (Figures 1 and 2). Her body mass index was 15 (weight: 35 kgs and height: 1.5 m). Other findings were unremarkable. Cerebrospinal fluid (CSF) analysis and electroencephalography (EEG) were both normal. On the basis of history, clinical presentations, and findings on neuroimaging, a diagnosis of Wernicke's encephalopathy was established. The patient was admitted to the hospital. The patient was treated with intravenous thiamine 100 mg in 100 mL of normal saline per day. Patient's confusion and nystagmus improved within 2 weeks. There was no improvement in ataxia, amnesia, confabulation, and disorientation even after 1 month of treatment. After 3 months of treatment, patient had confabulations and amnesia. However her MMSE (attention, memory, and language) improved from 16 to 24 over the next 3 months. Further follow-up of the patients could not be done, as we lost the patient subsequently.

## 3. Discussion

Anorexia nervosa is an eating disorder, characterized by intense fear of gaining weight and refusal to maintain body weight at or above 85% of the average weight for his or her age and height [10]. Anorexia nervosa causes various medical complications [7]. WE is one rare complication out of them. 38 percent of anorexia nervosa patients have deficiency of thiamine [5]. To complicate matters, WE is misdiagnosed in anorexia nervosa patients, probably due to nonspecific clinical presentation [5]. Anorexia nervosa is quite rare in this part of the world (India). However new cases of anorexia nervosa are emerging, probably due to changing life style. The prevalence of eating disorders in India over a period of 6 years was 1.25% and there were few cases of anorexia nervosa. There are no prevalence studies on anorexia nervosa in India [11]. Our patient developed WE probably due to self-imposed long-lasting nutritional deprivation. In our case, the patient presented with confusion and ataxia and then on further evaluations had symptoms of amnesia, confabulation, nystagmus, and ptosis. The radiological findings on MRI brain in patients with Wernicke's encephalopathy include lesions in mammillary bodies, thalamus, tectal plate, and periaqueductal area. These radiological findings represent typical lesions characteristic of WE [1]. The diagnosis of Wernicke-Korsakoff syndrome was established based on the history, clinical findings, and MRI findings. Self-imposed long-lasting nutritional deprivation is thought to be the main cause of thiamine deficiency and subsequent encephalopathy. But adjunct factors such as magnesium depletion might also have played an important role in the development of Wernicke-Korsakoff syndrome. Our patient had no history of alcohol intake or other significant medical histories, which could be a risk factor for development of WE. The patient, however, had history of prolonged dieting, leading to severe malnutrition. The patient developed hypokalemia (2.2 meq/L) and hypomagnesemia (0.43 mg/dL), which could be the predisposing factors for progression of WE to Korsakoff's syndrome. Our assumption is in accordance with other studies [12]. Magnesium is a cofactor that plays an important role in catalytic action of thiamine pyrophosphokinase and converts thiamine into thiamine pyrophosphate. Hypomagnesaemia further depletes stores of thiamine [13]. In our patient the progression of WE to KS occurred, in spite of adequate doses of thiamine. This could be probably explained by the delay in diagnosis and treatment which puts the patient at high risk [14].

The patient was treated by conventional doses of thiamine 100 mg/day intravenously. However it is said that these patients require 500 mg/day of thiamine intravenously [14]. It is also plausible that brain damage could have been already present in the patient before treatment with thiamine began. Brain damage occurs as a complication in patients of anorexia nervosa [12].

Clinicians should have knowledge and awareness regarding this preventable neuropsychiatric syndrome. They should be vigilant when new sign and symptoms arise in patients with known psychiatric disorders, as these can be potential of this condition. Mental health professionals should ask history regarding the eating patterns of patients and thoroughly review the medical records to look for signs of nutritional deficiencies. Clinicians can miss the life-threatening condition of WE, if they think that patients symptoms can only result from psychiatric disorders and not from nutritional deficiencies. Further delay in treatment of WE, which is a reversible condition, can result in brain damage and cognitive sequelae.

## Figures and Tables

**Figure 2:**